# Male blue wildebeest increase activity during the rut, but not at the expense of rest

**DOI:** 10.1007/s00360-023-01493-6

**Published:** 2023-04-28

**Authors:** Illke B. Malungo, Nadine Gravett, André Ganswindt, Paul R. Manger

**Affiliations:** 1 School of Anatomical Sciences, Faculty of Health Sciences, University of the Witwatersrand, 7 York Road, Parktown, Johannesburg, 2193 South Africa; 2 Endocrine Research Laboratory, Department of Anatomy and Physiology, Faculty of Veterinary Science, University of Pretoria, Soutpan Rd, Onderstepoort, Pretoria, South Africa; 3 Department of Zoology and Entomology, Faculty of Natural and Agricultural Sciences, Mammal Research Institute, University of Pretoria, Lynnwood Rd, Hatfield, Pretoria, South Africa

**Keywords:** Actigraphy, Activity patterns, Body temperature, *Connochaetes taurinus*, Faecal androgen metabolites, Sleep

## Abstract

Rest is a state of adaptive inactivity that increases the efficiency of activity by regulating its timing and reducing energy use when activity is not beneficial. Thus, animals can go without rest when specific demands, such as mating, favour being awake. Sexually active male blue wildebeest (bulls) are typically territorial, and it has been reported that when a bull is protecting a harem during the mating season (rut), he neither eats nor rests. We examined the daily activity and inactivity patterns of dominant bulls by means of actigraphy for 3 months, which included the rut. We also measured faecal androgen metabolite (fAM) levels and subcutaneous temperature, both of which have variances known to delineate the rut. During the rut, wildebeest bulls experienced higher levels of activity, fAM, and a greater daily range of subcutaneous temperature. Despite previous reports, the male blue wildebeest rested daily during the rut, and while the amount of rest was low, it was not substantially lower than prior to the rut. The amount of time spent inactive increased substantially after the rut. The timing of daily activity and inactivity patterns did not vary substantially across the recording period. Across the recording period, the average daily ambient temperatures decreased (seasonality), and the subcutaneous temperature followed this pattern, although it was not as marked. It appears that in the post-rut period a substantive increase in time spent at rest occurs, potentially allowing the wildebeest bulls time to recover following a period of intense activity.

## Introduction

For a few weeks each year in the southern African sub-region, sometime in the period of April to June depending on environmental conditions coinciding with the end of the rainy season, the blue wildebeest enter their breeding season, also known as the rut (Estes 1969). Sexually active male blue wildebeest (bulls) are typically territorial, and defend their territories with ritualized threat displays that, when not sufficient to deter an intruder, may develop into horn-sparring or head-butting contests (Estes 1969). Territorial defence becomes more intense during the rut, with the wildebeest bulls forming an exploded lek system, where they vigorously defend their harems and territories (the size of which is dependent on population density, Talbot and Talbot 1963). It has been reported that when a wildebeest bull is protecting a harem in his territory during the rut, he neither eats nor rests (Estes 1997). This would, however, imply that the most successful sexually active territorial bulls would need to go without rest (including sleep) for extended periods.

Sleep is a physiological and behavioural imperative that normally occurs daily, but thus far no function that can explain the huge variation in total sleep times observed across species has been identified (Lesku et al. 2012; Siegel 2012). Sleep may be viewed as a state of adaptive inactivity that increases the efficiency of activity by regulating its timing and reducing energy use when activity is not beneficial (Lesku et al. 2012; Siegel 2012). This means that animals can go without sleep when specific demands, such as mating, would favour being awake (Lesku et al. 2012; Siegel 2012). Lesku et al. (2012) showed that male pectoral sandpipers (*Calidris melanotos*) can maintain high neurobehavioral functioning, even though the time spent sleeping during a 3-week period of intense male–male competition for access to fertile females is greatly reduced; however, sleep loss appeared to be compensated for by a greater sleep intensity (Lesku et al. 2012). In a different context, great frigatebirds (*Fregata minor*) can minimize the amount of sleep during long flights, reducing total sleep time from 12.8 h per day down to 0.7 h per day (Rattenborg et al. 2016). Such observations are yet to be made in mammals, but the sexually active territorial wildebeest bulls during the rut appear to be potential candidates that may substantially lower, or even eliminate, the time spent at rest (presumably some portion of which would be sleep) during the mating season or rut (Estes 1997).

To investigate this possibility, we examined daily activity and inactivity patterns in four dominant male blue wildebeest by means of actigraphy (e.g., Davimes et al. 2016) for a period of 3 months, which included the time of the rut. In addition, we measured faecal androgen metabolite levels (Ganswindt et al. 2002; Hodges et al. 2010) and subcutaneous temperature (Kortner and Geiser 1994; Grigg et al. 2009), both of which have variances known to delineate the rut in other mammalian species.

## Materials and methods

### Experimental animals and site location

All animals were treated according to the guidelines of the University of the Witwatersrand Animal Ethics Committee (approval AESC number 2015/09/39/C), which parallel those of the National Institutes of Health (NIH) for the care and use of animals in scientific experimentation. Permission and permits to undertake the research were granted from the Gauteng Department of Agriculture and Rural Development, Division of Nature Conservation, and the Dinokeng Game Reserve, South Africa. Four free-roaming adult male blue wildebeest, which were identified as the dominant bull in their respective herds, due to their body mass, behaviour toward other individuals, and their subsequent defence of a territory during the rut, were located opportunistically in the Dinokeng Game Reserve. Each male had an estimated body mass of approximately 250 kg and were estimated to be between 8 and 15 years in age. All animals were judged to be in good condition by an experienced wildlife veterinarian. Dinokeng Game Reserve, situated in the north-eastern part of the Gauteng Province of South Africa (latitude° S: 25.403236; longitude° E: 28.296906), encompasses nearly 18 500 ha. The grassland and bushveld bioregions support an abundance of wildlife, with Dinokeng Game Reserve housing many large herbivorous and carnivorous mammal species. The climate in this region can be described as warm and temperate, and the area experiences summer rainfall patterns between November and February in the form of thunderstorms that can be variable and erratic (Rutherford et al. 2006).

### Recording period and environmental conditions

The period during which the recording of activity, subcutaneous temperature, and the measurement of faecal androgen metabolite levels (see below) took place was from February 15 to May 11, 2016. During the recording period, the subject wildebeest had access to the entire Dinokeng Game Reserve, being allowed to freely roam wherever they chose. The average temperature for the recording period was 21 °C, with a mean maximum temperature of 39 °C, a mean minimum temperature of 1 °C, and an average daily rainfall of 0.07 mm. These weather data were recorded at the Pienaars River weather station and provided by the Agriculture Research Council (ARC) of South Africa. Sunrise times for the recording period ranged from 05:52 (February 15) to 06:36 (May 11), and sunset times from 18:50 (February 11) to 17:31 (May 11). Sunrise and sunset times were obtained from freely accessible online databases.

### Activity and temperature data loggers

Activity was logged in each animal using two subcutaneously implanted actiwatches, one the side of the neck and one on the upper hindleg. The actiwatch is a wristwatch size device that is commonly used for measuring sleep in humans (e.g., Ancoli-Isreal et al. 2003; Shambroom et al. 2011). Within each actiwatch is a piezoaccelerometer device connected to a microchip that sums and records the number of acceleration events per minute. The Actiwatch Spectrum (Philips Respironics) was used in the current study. These devices have a mass of 25 g and approximate dimensions of 35 × 35 × 12 mm. Each actiwatch was calibrated and programmed (data acquisition rate set at 1 min intervals, sampling rate of 32 Hz, sensitivity of 0.025 G) with the Philips Respironics Actiware 5 software, prior to implantation. Subcutaneous temperature was logged using an iButton subcutaneously implanted with the neck actiwatch in each animal (see below). The iButton is a microchip enclosed in a round, stainless steel case and has a diameter of approximately 16 mm and a depth of 5 mm. The iButton thermochron hi-resolution (DS 1922L, Fairbridge Technologies) is a multi-use temperature data logger with a range of − 40 to 80 °C and measuring accuracy of ± 0.5 °C. The sampling frequency for each iButton was set to 30 min with the Cold Chain ThermoDynamics software (version 4.9). Prior to implantation, the wristbands were removed from the actiwatches and all actiwatches and iButtons were wrapped with standard electrical insulation tape and then covered in two coats of biologically inert wax (SasolWax 1276, Sasol, Johannesburg, South Africa). The devices were then placed in a container with formalin pellets for sterilization for a minimum of 24 h prior to the implantation procedure.

### Surgical implantation and removal of actiwatches and iButtons

The four blue wildebeests were darted and immobilized with weight appropriate doses of thiofentanyl (4 mg of A30–80^®^, Wildlife Pharmaceuticals, South Africa) and azaperone (40 mg Stresnil^®^, Janssen Pharmaceutica, South Africa). Following immobilization, the animals were placed in sternal recumbency, respiration and body temperature were monitored, and they received oxygen via a nasal tube throughout the procedure. The two implantation sites (the side of the neck and hind-leg) were shaved, cleaned, and disinfected with chlorhexidine (Kyron, South Africa). Sterile drapes were clamped in place over the implantation sites to isolate the cleaned areas. A small incision (approximately 4 cm in length) was made at each of the implantation sites and a subcutaneous pocket extending approximately 10 cm from the incision sites was created. The sterilized actiwatches were rinsed in sterile saline and inserted into each pocket. A sterilized iButton was rinsed in sterile saline and placed into the pocket created on the side of the neck in addition to the actiwatch. The incision sites were sutured closed by means of intradermal and superficial stitches and sterilized with the topical antiseptic spray Necrospray^®^ (Centaur Labs, Johannesburg, South Africa). Following the surgical procedure each animal was fitted with a radio transmitter collar (African Wildlife Tracking, Pretoria, South Africa) and plastic ear tags for individual identification (yellow, green, blue, and white) and then received weight appropriate doses of antibiotics and analgesics (8 ml of Draxxin^®^, Zoetis and 9 ml of Ketofen®, Zoetis). A radio transmitter collar was placed on each of the animals to monitor their recovery post-surgery and to relocate them on a weekly basis (to collect faecal samples) and after the 3-month recording period (to retrieve the implants). To reverse the anaesthesia, each animal was injected with weight appropriate doses of naltrexone (50 mg of Trexonil^®^, Wildlife Pharmaceutical, South Africa) and was monitored until they were able to stand unassisted and move off freely. Total time for the entire surgical procedure was less than 1 h, from the time the animal was darted to the time the reversal drugs were administered.

Following the recording period, the implanted animals were located and immobilized in the same manner as described for the implantation. Once the animal was immobilized the radio collar was removed and the implant sites were once again closely shaved and disinfected, and the devices retrieved. The devices were located in the same position as when implanted, and a small amount of scarring was observed around each of the implants. The amount of scarring was similar across all implants and was not to such a degree that raised concerns about the scarring preventing movement detection by the devices. The incisions were sutured closed and sterilized by spraying liberally with a topical antiseptic spray (Necrospray^®^, Centaur Labs, South Africa). The animals received weight appropriate doses of antibiotics (20 ml Lentrax^®^, Merial, South Africa) and the reversal drug, naltrexone (40 mg Trexonil^®^, Wildlife Pharmaceuticals, South Africa). No animals exhibited any ill effects of the implantation procedure, and all were in good health following the recording period.

### Faecal sample collection

Faecal samples were collected from each of the four male blue wildebeest at least once a week for the duration of the experimental period, with the animals being located using radio telemetry. The coloured ear tag for each individual was noted, and they were then observed through binoculars from a distance of approximately 20 m until they defecated (the animals were accustomed to being observed by people from vehicles in the game reserve and were thus not disturbed). The researchers then alighted from their vehicles, noting landmarks near the defecation site, and rapidly found (within 1 min) the recently defecated sample visually and cross-checked this with warmth and odour to differentiate this faecal matter from others in the vicinity. The animals slowly moved off when the researchers alighted from the vehicle. In addition to the four males, faecal samples were also collected from randomly selected juveniles (*n* = 20) of unknown sex for assay validation purposes. A total of *n* = 51 samples was collected. For each sample, approximately 40 g of fresh faecal matter was collected in a plastic container as soon after defecation as possible and placed immediately on ice in the field and subsequently stored at − 20 °C until analysis. Faecal samples were taken from the centre of the stool to avoid any cross contamination with urine, soil or water.

### Faecal steroid extraction and analysis

Frozen faecal samples were lyophilized and pulverized using a metal strainer allowing the removal of coarse material. Following this, 0.10–0.11 g of faecal powder was mixed with 3 ml of 80% ethanol and the suspension vortexed for 15 min. Subsequently the samples were centrifuged for 10 min at 1500 × g. Supernatants were transferred into micro-centrifuge-tubes and stored at − 20 °C until analysis (Scheun et al. 2017).

All steroid extracts were measured for immunoreactive faecal androgen metabolite (fAM) concentrations using an enzyme immuno assay (EIA) first described by Palme and Möstl (1993), which had been used to reliably monitor fAM alterations in other mammals (Ganswindt et al. 2010; Medger et al. 2018). The EIA used an antibody against testosterone-3-CMO-BSA. Further assay characteristics, including a full description of the assay components and cross-reactivities, is provided by Palme and Möstl (1993). The EIA was biologically validated for blue wildebeest by comparing fAM concentrations from individuals at different maturation stages, with significantly higher fAM concentrations found in adult males 0.71 ± 0.33 µg/g dry weight (DW) (mean ± SD) compared to juveniles (0.46 ± 0.33 µg/g DW) (*t*_31,20_ = 2.67; *p* = 0.01). The assays were performed on microtiter plates according to the protocol described by Ganswindt et al. (2002). Intra- and Interassay coefficients of variation (CV), determined by repeated measurements of high and low value quality controls were 5.0% and 5.1% (intra-assay CV), and 11.1% and 15.6% (inter-assay CV), respectively. The sensitivity of the EIA used was 18 ng/g. All faecal samples were prepared and analysed in the Endocrine Research Laboratory, Faculty of Veterinary Science, University of Pretoria. The individual fAM concentration for each focal animal for the 3-month recording period was pooled to obtain the overall average for the group.

### Organization of data for analysis

**Fig. 1:**
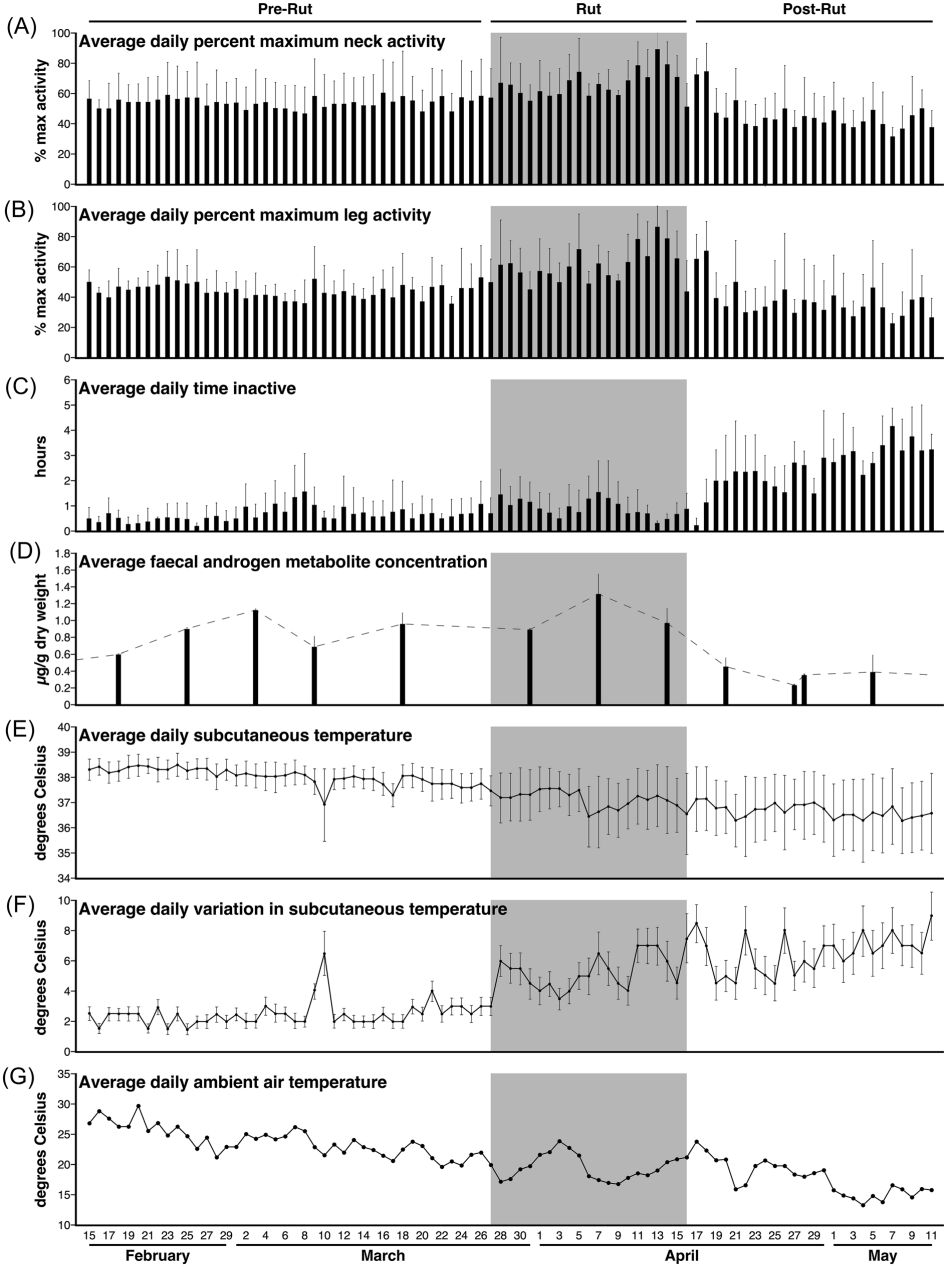
Average daily percentage maximum of neck (**A**) and leg (**B**) activity, the average daily time inactive (**C**), the average faecal testosterone levels (**D**), the average daily subcutaneous temperature (**E**), the average daily variation in subcutaneous temperature (**F**) of the blue wildebeest and the average daily ambient air temperature in Dinokeng Game Reserve (**G**). The graphs represent these different factors across the 3-month recording period, with each bar/point representing each day (dates provided in graph **G** and apply to all), including its standard deviation for the entire recording session. The three distinct periods within the recording session, Pre-rut, Rut, and Post-rut are indicated at the top of graph **A**, with the shaded area in each graph showing the rut period specifically. Graphs **A** and **B** show the average daily percentage maximum for the neck and leg activity, respectively. Note the increase in the average neck and leg activity during the rut. Graph **C** shows the average daily time spent inactive measured in hours. Note the increase in the time spent inactive during the post-rut period. Graph **D** shows the average faecal androgen metabolite concentrations for the group of 4 blue wildebeest. Note the peak in testosterone during the rut period and the decline in the post-rut period. Graph **E** shows the average daily subcutaneous temperature. Note the steady decline of the daily subcutaneous temperature over the 3-month recoding period. Graph **F** shows the average daily variation in the subcutaneous temperature (the daily maximum subcutaneous temperature minus the daily minimum subcutaneous temperature) for the recording period. Note the increase in the variation during the rut and post-rut period. Graph **G** shows the average daily ambient air temperature of the 3-month recording period in Dinokeng Game Reserve. Note the steady decrease in temperature associated with the changing seasons

**Fig. 2:**
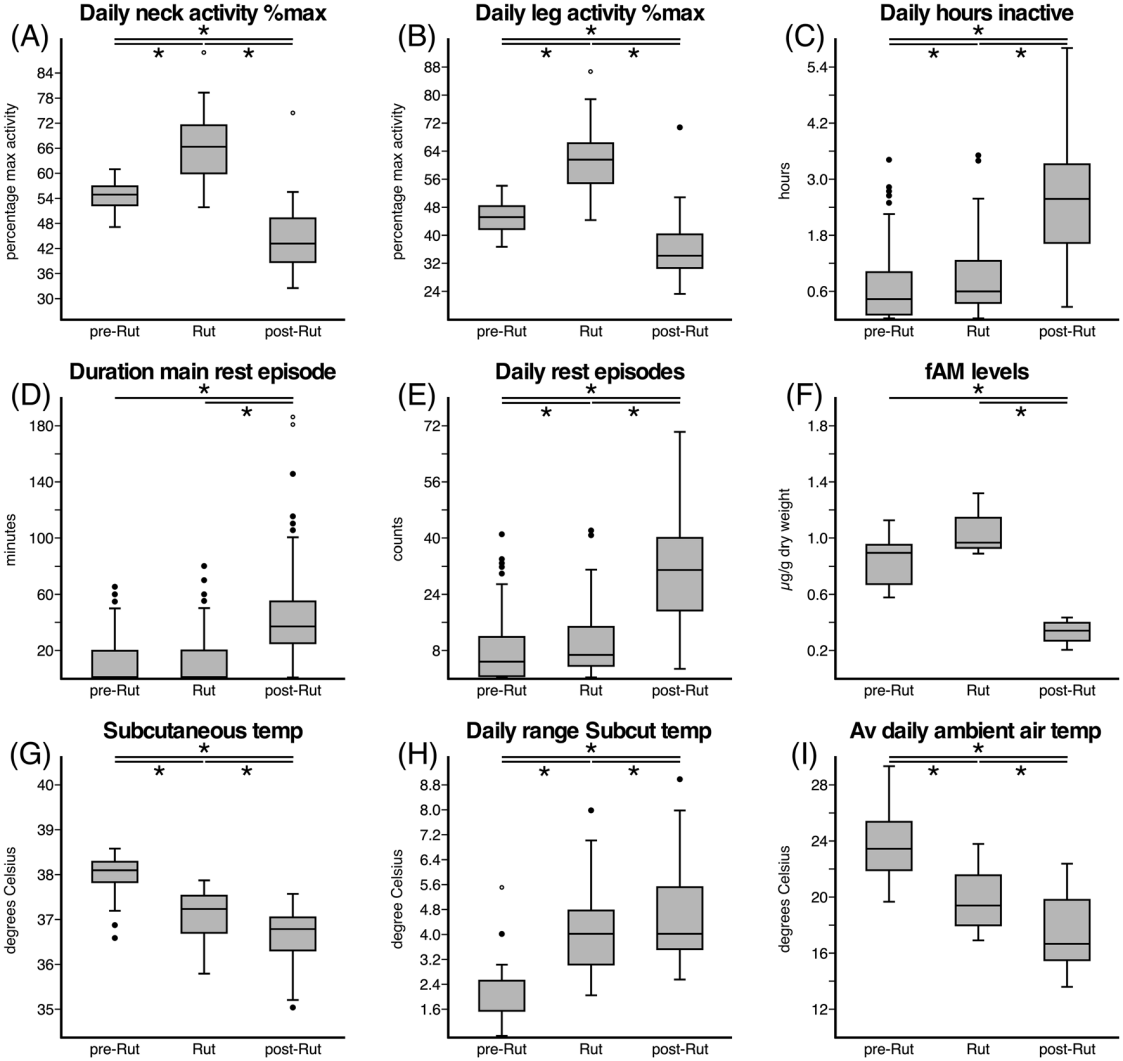
Box and whisker plots showing the distributions of group averages of daily counts of percentage maximum neck (**A**) and leg (**B**) activity, the daily time inactive (**C**), the duration of main rest episode (**D**), the number of daily rest episodes (**E**), the faecal androgen metabolite concentration (fAM) (**F**), the daily subcutaneous temperature (**G**), the daily variation in subcutaneous temperature (**H**) of the blue wildebeest, and the daily ambient air temperature in Dinokeng Game Reserve (**I**) during the three periods, Pre-rut, Rut, and Post-rut. *Indicates statistically significantly difference, °indicates outliers, •indicates extreme values. Note the statistically significant increases in neck and leg activity during the rut period (**A**, **B**), the increase in inactivity in the post-rut period (**C**), the increased length of the main rest episode in the post-rut period (**D**), and the increase in the number of rest episodes in the post-rut period (**E**). Moreover, the rut is marked by an increase in the levels of faecal testosterone (**F**) and a greater range in daily subcutaneous temperature (**H**). *Av* average, *Subcut* subcutaneous, *temp* temperature

The data presented herein are the grouped data for all four individual wildebeest, with results provided throughout as the mean ± the standard deviation (SD). The data were combined into group data as all individual wildebeest showed similar patterns of activity, inactivity, subcutaneous temperature, and fAM levels, with individual data not contradicting the group patterns described; thus, for brevity, only the group data are presented. The data were analysed for the 3-month recording period and for the average 24-h period. The data were then divided into three periods (pre-rut, rut, and post-rut) for further analyses. The rut was delineated by the highest peak in the average fAM level in combination with other factors described in the results section (see also Figs. 1, 2). Pre-rut is the time-period before the rut and post-rut is the time-period after the rut. The data recorded on the first 4 days following implantation were excluded from any analysis, allowing time for the animals to recover from any physical and chemical complications of the surgical procedure.

### Activity analyses

Phillips Respironics Actiware 5 was used to retrieve the recorded data from each of the implanted actiwatches. The raw data were exported to Microsoft Excel for analysis and the raw counts of each of the individual actiwatches were summed for each day. For each individual actiwatch, the highest summed activity count was recorded as maximum, or 100%, activity. The remaining counts for each actiwatch for each day were then converted to a percentage of the maximum activity. This process accounted for inter-device variability and sensitivity of the raw activity counts, allowing for unbiased comparisons between the individual actiwatches and between animals. For statistical comparisons 21 day pre-rut, rut, and post-rut, were compared to determine whether differences in activity levels between the three distinct periods occurred. Thus, data for percentage maximum activity of each neck actiwatch and each leg actiwatch on each day were pooled for the four animals (giving 84 data points for neck activity for each period, pre-rut, rut, and post-rut). These pooled data were averaged and used to determine the levels of daily activity for the group between the recording periods as well as being compared statistically between periods.

### Inactivity analyses

Data obtained from the neck and leg actigraphs were scored concurrently in 1-min epochs as either active or inactive. For an epoch to be scored as active either the neck or leg actigraphs had to have an activity score greater than zero. Inactive epochs were scored when both the neck and the leg actigraphs had an activity score equal to zero. From the 1 min scored data, the modal state for 5 min was calculated and used to determine, for each animal, total inactive time each day, number of inactive episodes each day and the average duration of inactive episodes. These data were then averaged across the individuals, daily, to determine group level changes across the entire recording session, and for statistical comparisons 21 day pre-rut, rut and post-rut, were compared to determine whether differences between the three distinct periods occurred. In addition, these data were used to determine the daily patterns of inactivity during the three distinct periods.

### Subcutaneous temperature

The iButton recordings were downloaded and analysed using ColdChain ThermoDynamics version 4.9 and exported to Microsoft Excel for further analysis. To determine average daily subcutaneous temperature, the 48 daily recordings from each of the four animals on the same day were summed and divided by 192 (48 recordings, 4 animals). For statistical comparisons, averages from 21-day pre-rut, rut, and post-rut, were compared to determine whether differences between the three distinct periods occurred. To obtain the daily pattern of variation in subcutaneous temperature, the time matched recordings from each animal across the 24-h period were summed and averaged across the pre-rut, rut, and post-rut periods (21 days per period, 4 animals). To determine the daily range of subcutaneous temperature, the lowest recorded temperature was subtracted from the highest recorded temperature for each animal for each day. These were then averaged for the group, daily, and plotted for the entire recording session to examine differences, or for statistical comparisons, averages from 21-day pre-rut, rut, and post-rut, were compared.

### Statistical analysis

To compare the effects of the different factors during the periods, pre-rut, rut, and post-rut, a one-way ANOVA (type III) was conducted and post-hoc (Tukey HSD) pairwise comparisons were used to determine statistically significant differences at the *p* < 0.05 level, with animals as a random independent variable, thereby accounting for the repeated measures obtained from each animal. To determine the relationships between the ambient air temperature, inactivity, and subcutaneous temperature, a multiple regressions and correlations test was conducted. The statistical analyses were conducted using IBM SPSS statistics software version 23.0 and PAST version 3.14 (Hammer et al. 2001).

## Results

The current study examined how the rut affects both activity and inactivity levels and timing in the blue wildebeest. Based on an increase in the levels of fAM (though not statistically significant, see below), corresponding with a major increase in the daily range of subcutaneous body temperatures recorded, and an increase in the amount of daily activity recorded in the neck and leg actiwatches, the blue wildebeest rut was determined to occur between the 27th of March and the 16th of April, 2016 (Figs. 1, 2). In contrast to these changes, the timing of daily activity and inactivity patterns did not vary substantially with the rut compared to the pre-rut and post-rut periods, although the amount of time spent inactive did increase significantly after the rut (Figs. 1, 2). Throughout the recording period, the average daily ambient temperatures decreased steadily, along with the change of season, and the subcutaneous temperature followed this pattern, although it was not as marked (Figs. 1, 2). Thus, the focal male wildebeest experienced higher levels of fAM, a greater range of body temperatures, and a higher level of activity during the rut, but this did not preclude them from having sufficient time each day to rest during the rut, although a major increase in the time spent resting after the rut was observed.

### Levels of faecal androgen metabolites prior to, during, and after the rut

Overall individual mean fAM level was 0.76 ± 0.25 µg/g DW during the pre-rut period, 1.04 ± 0.23 µg/g DW during the rut, and 0.36 ± 0.11 µg/g DW during the post-rut period (Fig. 2F). There was a significant difference in fAM levels between the three periods (ANOVA, *F *(2; 9) = 14.03, *p* = 0.002), with significantly lower fAM levels during the post-rut period compared to the pre-rut (Tukey’s HSD, *p* = 0.008) and the rut period (Tukey’s HSD, *p* = 0.002) (Fig. 2F). Levels of fAM did not differ significantly between the pre-rut and rut period (Tukey’s HSD, *p* = 0.325).

### Activity levels prior to, during, and after the rut

In the pre-rut period, the average daily percentage of maximum neck activity was 54 ± 16%, while that for the leg was 45 ± 12% (Fig. 2). During the rut these counts increased significantly, with the daily percentage of maximum neck activity being 67 ± 16% and leg activity being 62 ± 18% during the rut period (Mann–Whitney, Bonferroni corrected, *p* < 0.001). During the post-rut period, the percentage of maximum neck and leg activity were both statistically significantly lower than both the rut and pre-rut (Mann–Whitney, Bonferroni corrected, *p* < 0.001), with the average daily percentage of neck activity being 45 ± 17%, while that of the leg was 37 ± 21%. Thus, the rut was the period with the greatest overall level of activity, while the post-rut was the period with the lowest overall level of activity (Figs. 1A–C; 2A, B).

### Timing of activity prior to, during, and after the rut

**Fig. 3:**
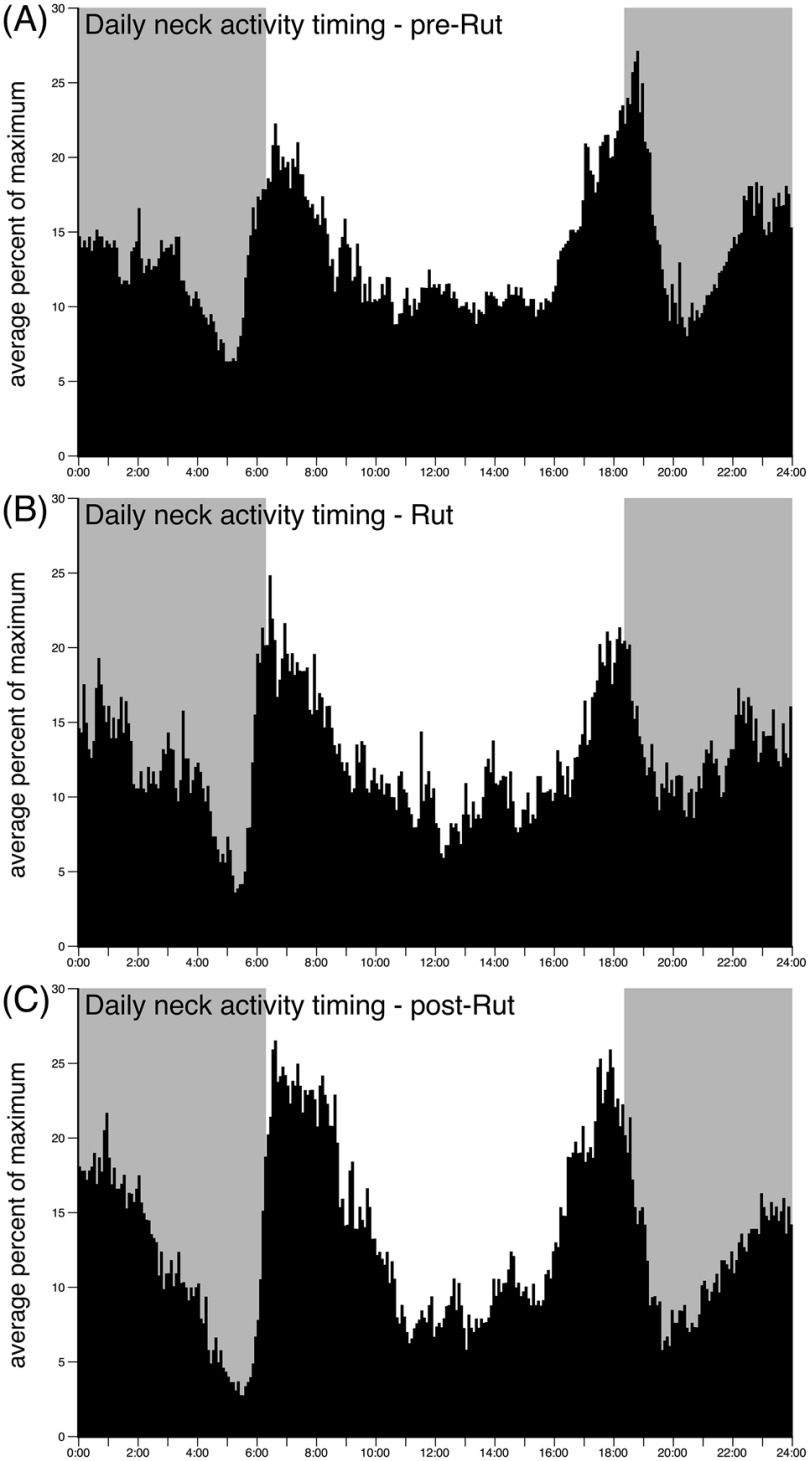
Average percentage maximum of the daily timing of neck activity for each 5 min over a 24-h period. Graphs **A**, **B** and **C** show the neck average percentage maximum for the pre-rut, rut, and post-rut periods, respectively. The shaded area indicates the period between sunset and sunrise. Each bar represents the 5-min average of the full group of animals over a 21-day period (i.e., each 5 min averaged for 21-day pre-rut, 21 days during the rut, and 21-day post-rut) over the 24-h period. Note the steady decrease in the levels of activity from midnight to pre-dawn, and the substantive matutinal and vespertine peaks. Although the general pattern of activity remains similar across the periods, the intensity of the pre-dawn decline in activity is more evident in the rut (**A**) and post-rut (**C**) periods

**Fig. 4:**
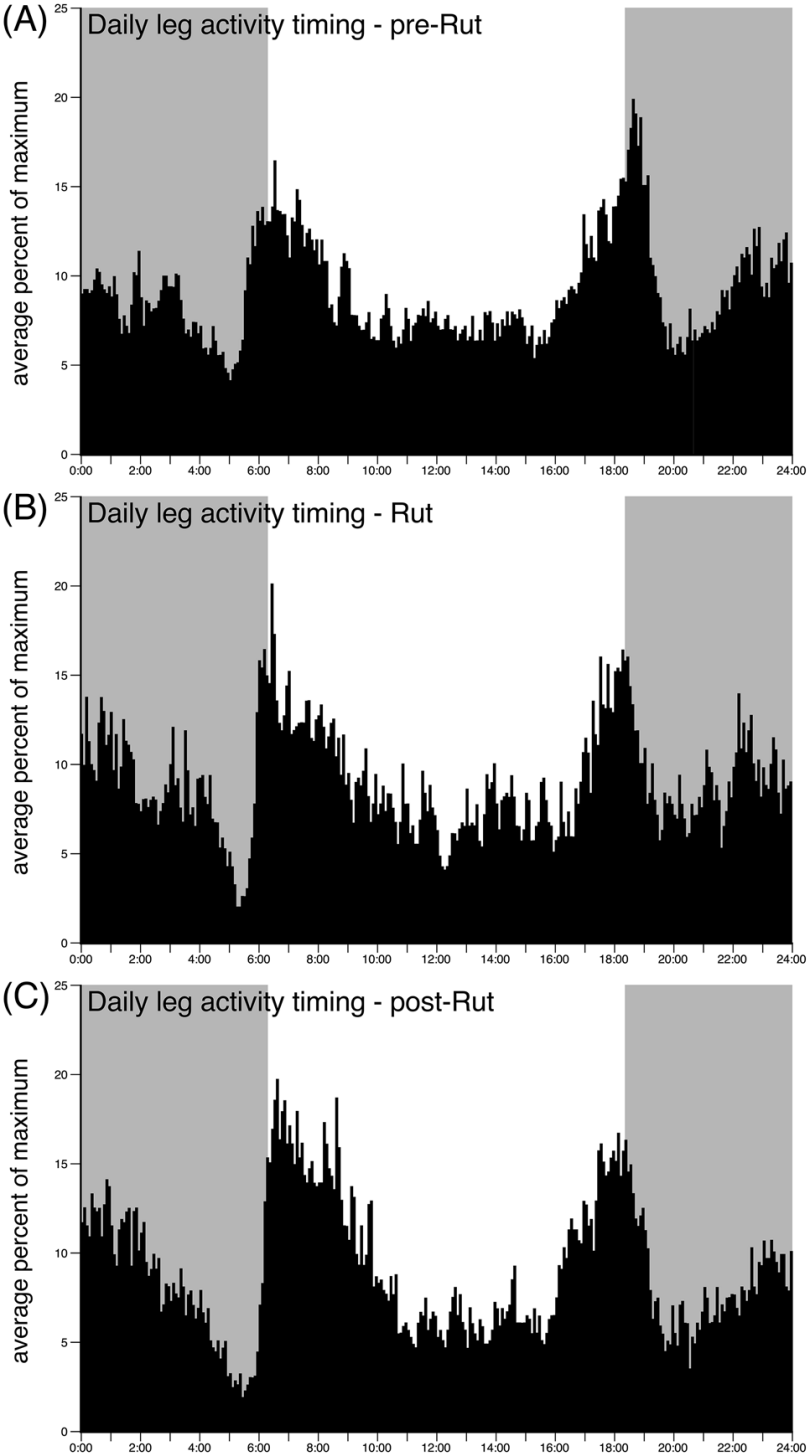
Average percentage maximum of the daily timing of leg activity for each 5 min over a 24-h period. Graphs **A**, **B** and **C** show the leg average percentage maximum for the pre-rut, rut, and post-rut periods, respectively. The pattern of activity is very similar to that seen for the neck (see Fig. 3). Conventions as in Fig. 3

Throughout the recording period, the four male blue wildebeest showed a similar timing of activity throughout the day (Figs. 3, 4). From midnight to the pre-dawn period there was a steady decrease in the levels of activity, with the pre-dawn period being the time of least activity. Around sunrise a substantive matutinal peak, lasting approximately 3 h, in activity was observed prior to a decline in activity levels throughout the day. Prior to, and around the time of sunset, a substantive vespertine peak, lasting approximately 3 h, in activity was observed, which was followed by a significant drop in activity levels prior to a steadily increasing level of activity until midnight. When compared across the three periods, pre-rut, rut, and post-rut, specific differences in the activity patterns were noted. While the midnight to pre-dawn decline in activity was present during all three periods, the intensity of this decline was more prevalent during the rut and even more so during the post-rut period, leading to a more noticeable pre-dawn nadir in activity. The matutinal and vespertine activity peaks did not change between the three periods, but the level of diurnal activity between these two major activity peaks was lower during the rut compared to the pre-rut period, and lower in the post-rut period compared to the rut. The post-vespertine dip in activity levels was less apparent during the rut than the pre- and post-rut periods, as was the steady increase in activity levels until midnight. Thus, while the general pattern of activity remains similar across the three periods, the relative amounts of activity at different periods throughout the day did change in subtle ways.

### Inactivity prior to, during, and after the rut

The average daily time spent inactive was 1.23 ± 1.24 h for the 3-month recording period. An ANOVA on the effects of the periods on the total hours spent inactive yielded a significant difference between the periods (*F* (2;6) = 42.47, *p* < 0.001). During the pre-rut period, an average of 0.64 ± 0.68 h per day was spent inactive. The average daily time spent inactive increased slightly during the rut to 0.88 ± 0.78 h, which was a significant increase compared to pre-rut inactivity levels (Tukey’s HSD, *p* = 0.044). During the post-rut period, the average daily time spent inactive increased to 2.57 ± 1.29 h, which was significantly higher than both the pre-rut period (Tukey’s HSD, *p* < 0.001) and the rut (Tukey’s HSD, *p* < 0.001) (Figs. 1C; 2C). Thus, the amount of time spent inactive, with much of this time presumably being spent in sleep, did not change by much between the pre-rut and rut, but increased distinctively, 1.92-fold (rut) and 2.95-fold (pre-rut), in the post-rut period.

For each day, the main rest episode was identified as the longest consolidated period of inactivity recorded in a 24 h period. During the pre-rut, rut, and post-rut periods the onset of the main rest episode occurred on average at 03h17 ± 1.88 h, 04h21 ± 1.42, and 04h05 ± 2.02 h, respectively, and had an average duration of 32.16 ± 14.16 min, 34.09 ± 17.21 min and 50.06 ± 32.80 min, respectively. The duration of the main rest episode was significantly different between the three periods (ANOVA, *F* (2;6) = 34.53, *p* = 0.001). Thus, in addition to resting more during the post-rut period, the average length of the main rest episode was significantly longer during the post-rut period compared to the pre-rut period (Tukey’s HSD, *p* < 0.001) and the rut (Tukey’s HSD, *p* < 0.001) (Fig. 2); however, no difference in the length of the main rest episode was observed between pre-rut and rut (Tukey’s HSD, *p* = 0.062).

On each of the recording days, multiple episodes of inactivity were observed, indicating that blue wildebeest, like most mammals, are likely to be polyphasic sleepers. Throughout the 3-month recording period, the average number of daily inactive episodes was 14.77 ± 14.83 During the pre-rut, rut, and post-rut periods, the average number of daily inactive episodes was 7.70 ± 8.22, 10.50 ± 9.39, and 30.89 ± 15.51, respectively. An ANOVA on the effect of the periods on the number of daily inactive episodes yielded significant variation between the periods (*F* (2;6) = 42.47, *p* < 0.001). The number of daily inactive episodes was significantly different between the pre-rut period and the rut (Tukey’s HSD, *p* = 0.044), as well as between the post-rut period and both the pre-rut period and the rut (Tukey’s HSD, *p* < 0.001 for both instances). Thus, the amount of time spent inactive indicates the possibility of an inactivity rebound period following the sustained increased levels of activity during the rut (see above). This increase in daily amounts of inactivity specifically coincided with the end of the rut (Fig. 1).

### Timing of inactivity prior to, during, and after the rut

Throughout the 3-month recording period, the majority of time spent inactive occurred during the night, with an average percentage of time spent inactive during the day being 16.69 ± 22.65% and 76.69 ± 30.17% during the night. During the pre-rut, rut, and post-rut periods the average percentage of time spent inactive during the day was 15.59 ± 25.49%, 18.93 ± 22.83%, and 16.26 ± 16.50%, respectively, and during the night it was 72.49 ± 36.49%, 77.5 ± 27.07%, and 83.74 ± 16.50%, respectively. Thus, during the post-rut period, more time was spent inactive at night than in the pre-rut and rut periods.

**Fig. 5:**
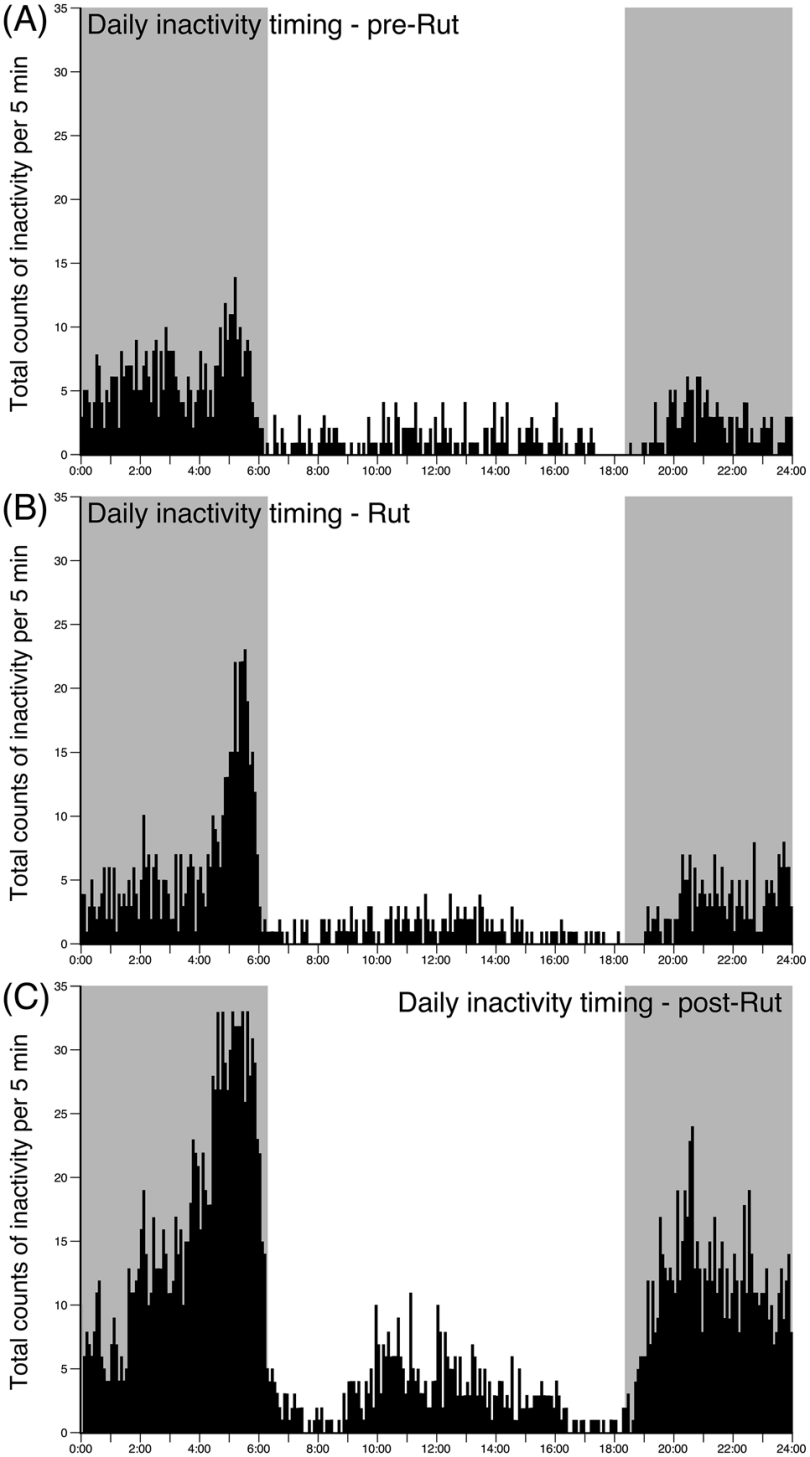
Graphs illustrating the total counts of inactivity for any given 5 min period scored over 21 days in the pre-rut (**A**), rut (**B**) and post-rut (**C**) for all animals. The grey regions represent the period between sunset and sunrise. Note that there was a clear pattern of the timing of inactive episodes observed throughout the recording period. The majority of inactive episodes were observed between midnight and sunrise, but during the rut (**B**) there was a distinct spike of inactivity between 04:00 and 06:00, while during the post-rut (**C**) this spike of inactivity was larger, but occurred over a longer time period, between 02:00 and 06:00

A clear pattern in the timing of inactive episodes was observed throughout the recording period (Fig. 5). The majority of inactive episodes were observed between midnight and sunrise, but during the rut, there was a distinct spike of inactivity between 04h00 and 06h00, while during the post-rut period, this spike of inactivity was larger, and occurred over a longer period, between 02h00 and 06h00. Between sunrise and sunset, the amount of inactivity was low, but there was a noticeable increase in inactivity between sunrise and sunset during the post-rut period. Between sunset and midnight, several inactive episodes occurred, but the number was far greater during the post-rut period compared to the pre-rut period and the rut (Fig. 5). These observations are consistent with the activity patterns described above, and the general increase in inactivity levels in the post-rut period compared to the pre-rut and rut periods. The very distinct pre-dawn spike in inactivity during the rut indicates that all the wildebeest recorded during this period were likely to be sleeping at this time of the day. Despite these variances, the general temporal pattern of inactivity did not differ between the three periods.

### Average daily subcutaneous temperature prior to, during, and after the rut

The average daily subcutaneous temperature showed a steady decline over the 3-month recording period, with temperatures being approximately 38.5 °C at the start of the recording period and 36.5 °C toward the end of the recording period, and the overall average being 37.43 ± 0.78 °C (Fig. 1E). An ANOVA showed a significant difference between the periods for average daily subcutaneous temperature (*F* (2;4) = 16.49, *p* = 0.012). During the pre-rut, rut, and post-rut periods the average daily subcutaneous temperature was 38.04 ± 0.36 °C, 37.12 ± 0.52 °C, and 36.64 ± 0.63 °C, respectively. This drop in subcutaneous temperature was significantly different between the pre-rut and rut periods as well as between the post-rut and both the pre-rut period and the rut (Tukey’s HSD, *p* < 0.001 for all instances) (Fig. 2G).

### Average daily variation in subcutaneous temperature prior to, during, and after the rut

**Fig. 6:**
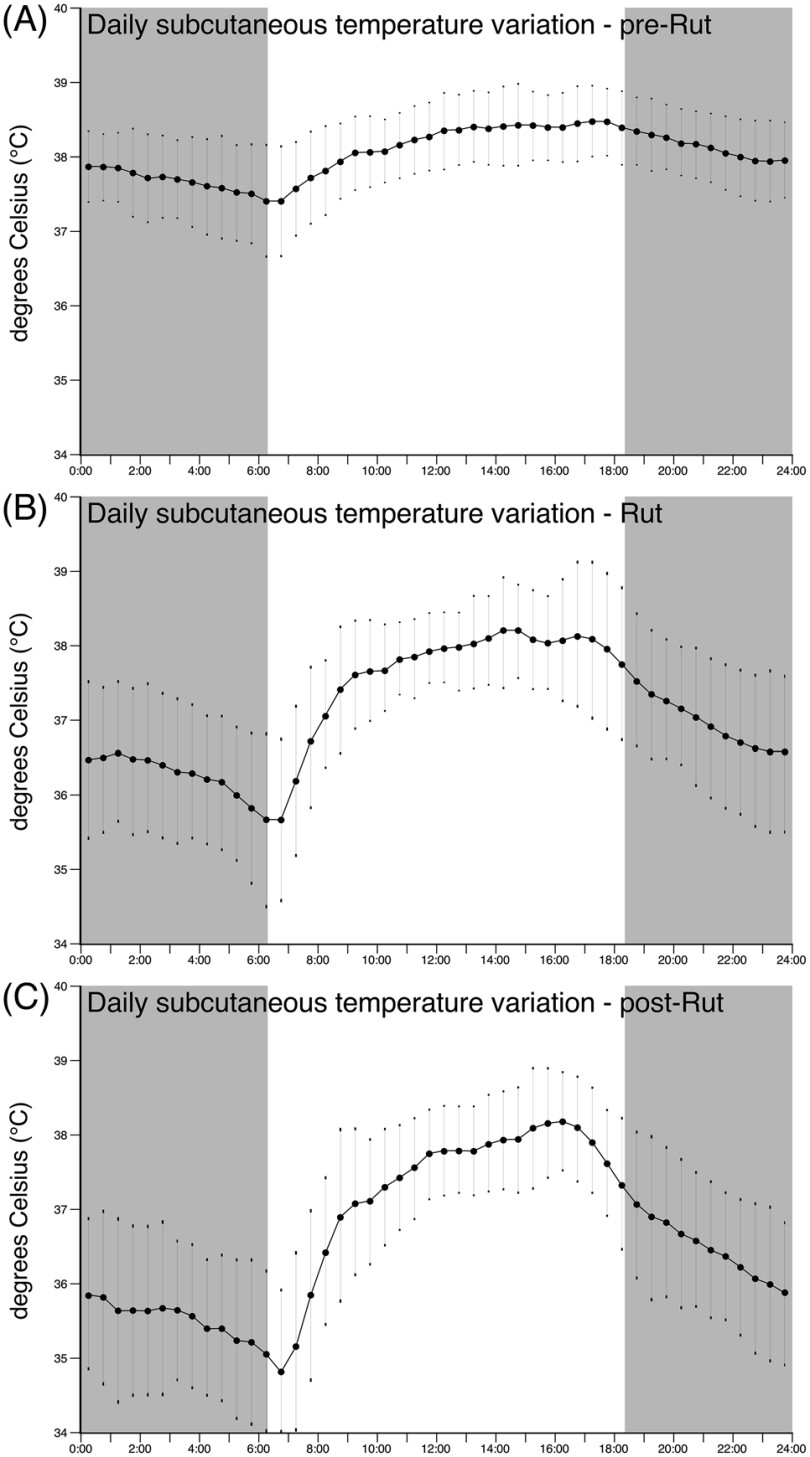
Daily pattern of subcutaneous temperature over a 24 h period, each 30 min recording averaged over all animals for 21 days in the pre-rut (**A**), rut (**B**), and post-rut (**C**). The grey regions represent the period between sunset and sunrise. Note the increased variance in the subcutaneous temperature reflected across the day during the rut (**B**) and post-rut (**C**) periods, with substantial dips in subcutaneous temperature observed around sunrise and sunset

Unlike the steady decline in average daily subcutaneous temperature, the average daily variation in subcutaneous temperature (the daily maximum subcutaneous temperature recorded minus the daily minimum subcutaneous temperature recorded), exhibited an increased variance during the rut and post-rut periods compared to the pre-rut period (Fig. 1F). There was a significant difference between the periods for the daily variation in the subcutaneous temperature (ANOVA, *F* (2;4) = 16.49, *p* = 0.029). During the pre-rut period, the average daily subcutaneous temperature range was 1.91 ± 0.82 °C and increased significantly to an average of 4.01 ± 1.35 °C (Tukey’s HSD, *p* < 0.001) during the rut (Fig. 2). During the pot-rut period the average daily subcutaneous temperature range increased to 4.49 ± 1.49 °C which was significantly higher compared to both the pre-rut period (Tukey’s HSD, *p* < 0.001) and the rut (Tukey’s HSD, *p* = 0.011) (Fig. 2H). This increased variance in the subcutaneous temperature is reflected across the day, with substantial dips in subcutaneous temperature observed prior to sunrise and following sunset, as well as a greater increase in subcutaneous temperature during the afternoon (Fig. 6). This increased range of daily subcutaneous temperatures specifically marked the beginning of the rut (Fig. 1F).

### Average daily ambient air temperature prior to, during, and after the rut

The recording period spanned the days from February 15 through to May 11, meaning that the recording period spanned the last 2 weeks of summer, and all but the last 2 weeks of autumn. As would be expected, the average daily ambient air temperature showed a steady decline during this period, although there was substantial variation (Fig. 1G). The average daily ambient air temperature over the 3-month recording period was 21.04 ± 3.72 °C. An ANOVA on the daily ambient temperature yielded significant variation among the periods (*F* (2;84) = 57.19, *p* < 0.001). During the pre-rut period, the average daily ambient air temperature was 23.80 ± 2.45 °C, which was significantly higher than the average recorded during the rut period, which was 19.77 ± 2.28 °C (Tukey’s HSD, *p *< 0.001). During the post-rut period, the average daily ambient air temperature dropped to 17.32 ± 2.58 °C, and this average was statistically significantly lower than that recorded during both the pre-rut (Tukey’s HSD, *p* < 0.001) and rut periods (Tukey’s HSD, *p *= 0.004) (Fig. 2I).

### Average daily ambient temperature and average daily time inactive

**Fig. 7:**
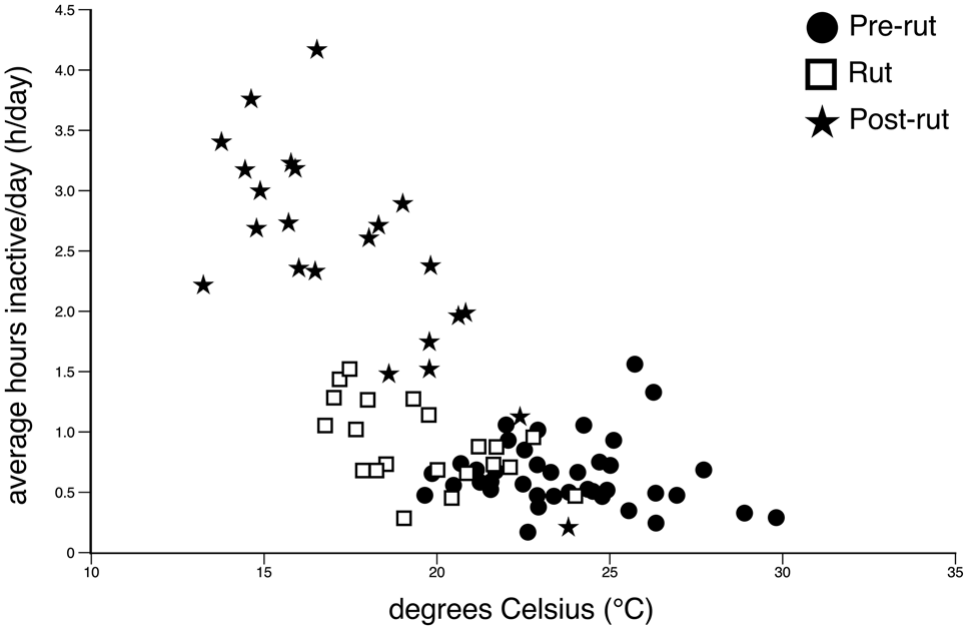
Graph showing the lack of a clear relationship between average ambient air temperature (°C) and the average hours of inactivity per day for the four male wildebeest recorded during the pre-rut (filled circles), rut (open squares) and post-rut (filled stars). The recording period covered the dates from 15 February (late summer) to 11 May (late autumn), with average daily temperatures dropping from close to 30 °C to around 13 °C. While there is clearly a trend for increased time spent inactive with the cooler weather, this trend is not strongly predictive

It is well-known that seasons affect the amount of rest/sleep experienced, generally with more rest/sleep during the cooler seasons (e.g., Borbély and Tobler 1996; Yetish et al. 2015), and as the recordings undertaken herein covered the dates from 15 February (late summer) to 11 May (late autumn), the effect of season on daily time spent inactive needs to be addressed. Over the recording period average daily temperatures dropped from close to 24 °C to around 17 °C. During the post-rut period, the period with the coolest average temperatures, the amount of inactivity was the highest, but when we compare across the entire recording period (Fig. 7), while we do see a trend toward increasing time per day spent inactive with cooler temperatures, this is a trend that does not have a strong predictive value. Rather, it appears that the major increase in daily inactivity during the post-rut period is driving this trend. While we cannot rule out completely that cooler temperatures play a role in the increased amount of time spent inactive over the recording session, any role would be minor in comparison with that driven by other factors, such as the rut, or recovery from the extensive activity during the rut.

## Discussion

In the current study, we used actigraphy to explore the assertion that during the rut, or breeding season, when blue wildebeest bulls maintain a harem in their territories, they neither eat nor rest (Estes 1997). The concept that the male blue wildebeest will not rest during the rut indicates that it is unlikely to spend any time asleep, despite sleep being one of the biological imperatives of mammalian biology (Mignot 2008). Our findings indicate that during the rut, the male blue wildebeest, while having significantly higher activity levels than either before or after the rut, do not increase the daily amount of time spent active at the expense of time spent at rest (potentially asleep). Following the rut there appears to be a significant increase for time spent resting, which may be regarded as a period where the male wildebeest recuperate following the high activity levels during the rut.

### Defining the rut

The wildebeest rut is a notoriously difficult period to a priori define accurately, with a variable beginning and ending that is related to changes in environmental conditions (Skinner and Chimimba 2005). In the present study, instead of attempting to define the period of the rut prior to undertaking the study, we decided to record various parameters over an extended period, allowing us to a posteriori define the rut period with greater precision. As with other mammalian species that exhibit a rut period (Geist 1965; Geist and Walther 1974; Poole 1987; Balmford et al. 1993; Carranza et al. 1996; Whittle et al. 2000; Mysterud et al. 2002), related androgen levels of the male wildebeest under study increased (by approximately 1.4 times, although this is not a statistically significant increase) during this time compared to the pre-rut period, and these levels decreased substantially after the rut (to almost a third of the level observed during the rut, this being a statistically significant decrease). The beginning of the rut was marked by three additional specific measures taken in the current study, these being a significant increase in the daily variation of subcutaneous temperature, and significant increases in the average daily levels of activity recorded by both the neck and leg actiwatches. The end of the rut was also defined by three additional specific measures, these being significant decreases in the average daily levels of activity recorded by both the neck and leg actiwatches, and by a significant increase in the amount of inactivity recorded. By correlating the results for the faecal androgen metabolite levels, daily activity levels, daily inactivity levels, and daily subcutaneous temperature variations, we were able to reliably define a period of 3 weeks, from the 27th of March to the 16th of April, as the 2016 rut period for the four male blue wildebeest studied. During this period, opportunistic behavioural observations made during the collection of faecal matter, revealed that the male blue wildebeest under study also displayed typical rut behaviour such as increased calling, foaming at the mouth and multiple attempts to mount receptive cows (Estes 2014). Thus, our a posteriori definition of the rut appears to be supported by several independent measurements, and provides confidence in the comparisons between the pre-rut, rut and post-rut periods made herein.

### Activity levels before, during and after the rut

Our observations indicate that the rut period was clearly the most active time throughout the recording period. Overall activity levels during the rut were 1.2–1.4 times higher than during the pre-rut period and 1.5–1.7 times higher than the post-rut period. Thus, in agreement with qualitative observations made regarding activity levels of the male wildebeest during the rut (Estes 1997), we find that quantitatively the 21-day rut period is the most active time for the male wildebeest. This appears to support the concept that that male wildebeest are protecting a harem within their territory (Estes 1997), and that due to the possibility of intruders the activity levels are consistently high during this time to prevent loss of breeding rights. Interestingly, during the 21 days following the rut, which we term the post-rut period, the activity levels were substantially lower than both the rut and pre-rut periods. This indicates that post-rut, the male wildebeest likely need substantially more rest for recovery after the period of intense activity during the rut.

### Inactivity levels before, during and after the rut

One of the central aims of the current study was to test whether, during the rut, the male wildebeest did not rest at all, as indicated by Estes (1997). In the current study we used a very strict definition of rest, this being that both the neck and leg activity meters, combined, had to show modal count of no activity (i.e., a combined activity score of zero for both actiwatches for at least 3 of 5 1-min periods) for a period of 5 min. This strict definition of rest, indicating no major or consistent movement of the animals for a 5-min period, likely means we are underscoring total rest, but does allow us to be confident in our quantitative conclusions regarding variations in the amount of rest. Contrary to what is qualitatively reported by Estes (1997), we find that during the pre-rut, rut, and post-rut, rest occurred daily. The amount of daily rest obtained by the male wildebeest in the pre-rut and rut was similar, and with our strict scoring criteria for inactivity, amounted to 40–50 min of daily inactivity. Thus, even during the rut, the blue wildebeest appeared to rest for the same amount of time each day as during the pre-rut period. The conclusion derived by Estes (1997) appears to be understandable from the perspective that this daily rest during the rut occurred mostly between 4 to 6 am, a time when observations of male wildebeest activity or inactivity are unlikely to have been made with any consistency.

Interestingly, in the post-rut period, the amount of daily rest increased substantially compared to the pre-rut and rut periods. We observed that the amount of rest in the post-rut period increased to approximately 2.5 h per day, which is around 3–4 times greater than that observed daily in the pre-rut and rut periods. This finding mirrors the lowered amount of activity observed in the post-rut period compared to the rut and indicates that the post-rut period appears to represent a time of the year when the male blue wildebeest are undergoing a sustained period of increased rest in response to the sustained period of increased activity leading up to and during the rut. The emphasis placed on this substantial increase in rest in the post-rut period is possibly tempered by the reduced photoperiod as well as cooler weather conditions observed during this time. These changes in the weather conditions may play a role in the increased amount of rest observed in the post-rut period; however, it is unlikely that weather conditions alone account for the major increase in daily rest time observed in the post-rut period and that indeed much of this increase in daily rest can be attributed to a period of prolonged recovery after a period of intense activity.

### Sleep and the rut

One of the central biological imperatives of mammalian existence is the need to undergo the physiological and behavioural process of sleep (Campbell and Tobler 1984; Tobler 1995; Siegel 2005). Lack of sleep can lead to a range of neural and behavioural complications (see review by Boonstra et al. 2007) that can compromise survival. Thus, the indication by Estes (1997) that the male wildebeest do not rest during the rut also indicates that they do not enter the physiological and behavioural process of sleep during the rut. As outlined above, the male wildebeest do rest, for around 50 min per day, during the rut, and it is likely that much of this time, if not more given the strict scoring criteria applied to rest in the current study, is spent in sleep. Thus, despite the overwhelming need to protect a territory, harem, and breeding rights during the rut, the male wildebeest appear to obtain sleep daily. This finding complements previous findings in birds, where during breeding season, total sleep time is reduced significantly for male pectoral sandpipers (Lesku et al. 2012), with a similar observation being made in great frigatebirds during long flights (Rattenborg et al. 2016), but both species do sleep, despite being under intense pressure not to sleep. Interestingly, in the male pectoral sandpipers, the reduced amount of sleep may be compensated for by a greater sleep intensity, allowing for the shortened daily sleep times (Lesku et al. 2012), and this may also apply to the male blue wildebeest. However, to determine whether this is the case in the wildebeest, polysomnographic recordings would be required during the pre-rut, rut, and post-rut, and this is presently beyond our technical capacities.

### Summary

The present study demonstrates that a variety of measures indicate that the male blue wildebeest do enter a specific and consolidated rut period that lasts for approximately 21 days. Despite previous qualitative reports (Estes 1997), the male blue wildebeest do rest daily during the rut, and while the amount of rest may be low, it does not appear to be substantially lower than in the period prior to the rut. It appears that in the post-rut period a substantive increase in rest occurs, potentially allowing the male wildebeest time to recover following a period of intense activity. The blue wildebeest appears to form an exploded lek system for mating, and thus may represent only a portion of the spectrum of variation in rest and sleep across artiodactyls that show this system of mating. Other artiodactyls, such as the Uganda kob (*Adenota kob thomasi*), which form an intense and localized classical lek system (Buechner and Roth 1974), may show reduced or absent rest during the breeding season. It would be of interest to use actigraphy to test these potential variations in the amount of daily rest obtained in lek systems of varying intensity and size. This would allow the determination of whether a true spectrum exists, or whether at a certain level of intensity, related to different lek systems, the biological imperative of sleep is put into abeyance for a specific time and the effect this may have on survival and reproductive success.

## Data Availability

Data used in this study are available on request.
